# Sexual jokes at school and students’ life satisfaction: findings from the 2017/18 Swedish Health Behaviour in School-aged Children study

**DOI:** 10.1186/s13104-021-05691-9

**Published:** 2021-07-26

**Authors:** Sara Brolin Låftman, Ylva Bjereld, Bitte Modin, Petra Löfstedt

**Affiliations:** 1 Department of Public Health Sciences, Centre for Health Equity Studies (CHESS), Stockholm University, 10691 Stockholm, Sweden; 2 Department of Behavioural Sciences and Learning (IBL), Linköping University, 58183 Linköping, Sweden; 3 Department of Public Health and Community Medicine, Sahlgrenska Academy, University of Gothenburg, Box 100, 40530 Gothenburg, Sweden

**Keywords:** Sexual jokes, Sexual harassment, School, Adolescents, Positive mental health, Cantril ladder

## Abstract

**Objective:**

In a previous study we demonstrated that the occurrence of sexual jokes in the class was associated with higher levels of psychological health complaints. Building on and extending these findings, the aim of the current study was to examine if exposure to sexual jokes at the student and at the class level was inversely associated with students’ life satisfaction. Data were derived from the 2017/18 Swedish Health Behaviour in School-aged Children (HBSC) study, with students aged 11, 13 and 15 years (n = 3710 distributed across 209 classes). Exposure to sexual jokes at the student level was captured by one item. Exposure to sexual jokes at the class level was calculated by aggregating this measure. The Cantril ladder was used to operationalise life satisfaction. Two-level logistic regression analyses were performed.

**Results:**

Students who were exposed to sexual jokes at school were less likely to report high life satisfaction (OR 0.38, 95% CI 0.27–0.53). An inverse association was found between the class proportion of students who were exposed to sexual jokes and students’ likelihood of reporting high life satisfaction, whilst adjusting for exposure to sexual jokes at the student level (OR 0.98, 95% CI 0.97–0.9994). The findings highlight the importance of promoting a school climate without sexual harassment.

**Supplementary Information:**

The online version contains supplementary material available at 10.1186/s13104-021-05691-9.

## Introduction

Sexual harassment may be verbal or non-verbal, and one type of verbal harassment is sexual jokes [1]. Verbal sexual harassment may be expressed in terms of, e.g., unwanted comments about one’s body [2], disturbing sexual propositions, sexually insulting epithets [3], and homophobic name-calling [4]. Although there is no clearcut definition of sexual jokes, the fact that some acts of verbal sexual harassments are framed as ‘jokes’ implies that any negative reactions on the part of the victim may be dismissed with reference to the lack of humour, thus making it difficult for victims to defend themselves [5].

Prior research has shown that exposure to sexual harassment is associated with higher levels of psychological problems among adolescents [1, 2, 4, 6–8]. Analyses based on the same data as the current study demonstrated that the occurrence of sexual jokes in the class was associated with higher levels of psychological health complaints even when adjusting for student level exposure to sexual jokes [1], suggesting that also those not directly targeted may be affected. On a similar note, sexual harassment in school has been reported to be perceived as problematic not only by students who are directly exposed, but also by many of the students who are non-exposed [9]. Less is however known about the potential individual and contextual effects of exposure to sexual harassment on adolescents’ positive mental health.

Psychological problems and positive mental health constitute distinct concepts. Whereas the former reflects a pathological perspective (i.e., a focus on adverse health), the latter instead reflects a salutogenic approach (i.e., a focus on good health rather than just the absence of health problems). Measures of positive mental health include, e.g., mental well-being, excellent self-rated heath, and high life satisfaction [10]. Life satisfaction has been identified as an important indicator of adolescent subjective well-being [11] and psychosocial health [12]. According to the Swedish Education Act [13], schools are required to work with both health prevention and promotion, i.e., to prevent health problems but also to strengthen the prerequisites for good health among their students. Hence, it is important to identify the social determinants of psychological health problems and of positive mental health alike. Both psychosocial working conditions and social relationships at school are key social determinants of adolescent health. With regards to social relationships, this is true for friendships and supportive relations but also for relations characterised by strain, harassment, and bullying (including exposure to sexual jokes). By building on and extending the findings reported in our previous publication on sexual jokes and psychological complaints [1], but here instead focusing on life satisfaction as an indicator of positive mental health, the aim of the current study was to examine if exposure to sexual jokes at the student and the class level was inversely associated with students’ life satisfaction.

## Methods

### Data and participants

The data were derived from the cross-sectional Swedish Health Behaviour in School-aged Children (HBSC) study of 2017/18, as part of a collaborative World Health Organisation (WHO) project. The study was performed with students in grades 5, 7, and 9 (ages 11, 13 and 15 years). A two-step cluster sampling was used. A random, nationally representative sample of schools across Sweden were drawn, and in each school one class was randomly drawn to participate. The students responded to the questionnaire with pencil and paper in the classroom. The total sample included 4294 students. The response rate at the school level was 47% and the response rate among students in the participating schools was 89% [1, 14]. Due to anonymity of the responding schools, we were not able to examine if there was any systematic bias in the school-level non-response. For the current study, we excluded 25 students who attended classes with fewer than 8 responding students (in order to ensure a robust measure at the class level) and 559 students with missing information on any of the study variables, resulting in a study sample of 3710 students in 209 classes (i.e. 86.4% of the original sample).

### Measures

Exposure to sexual jokes at the student level was captured by one statement concerning bullying at school: “Other students have exposed me to sexual jokes”. Students who responded that this had happened “2 or 3 times a month” or more often were defined as exposed to sexual jokes at school*. *Exposure to sexual jokes at the class level was calculated by aggregating this measure to the class level, and was measured in per cent. Both measures have been used previously [1].

*Life satisfaction* was measured by the 11-step Cantril ladder. The respondents were given the following instructions: “Here is a picture of a ladder. The top of the ladder ‘10’ is the best possible life for you and the bottom ‘0’ is the worst possible life for you. In general, where on the ladder do you feel you stand at the moment? Tick the box next to the number that best describes where you stand*.*” [11]. Due to the skewed distribution, with a majority of students reporting higher scores, a dichotomous measure was constructed with scorings 0–5 classified as low life satisfaction and 6–10 as high life satisfaction. According to Due et al. [11], most previous studies based on HBSC data used this cut-off point (e.g., [12, 15–17]), although some have (also) used the cut-off point 0–8 vs. 9–10 (e.g., [11, 12, 17]), or the continuous measure [15].

Gender (with the categories boys and girls) and grade (containing grades 5, 7, and 9) were included as control variables at the student level. Prior analyses of the data showed that exposure to sexual jokes was more common among older girls than among younger ones, whereas there was no difference across grades among boys [1, 14]. Analyses of the same data have also shown that boys were more likely to report high life satisfaction than girls, as were older students compared with younger ones [17]. Since socioeconomic disadvantage and not living with two parents have been shown to be linked with a higher likelihood of exposure to sexual harassment [3] and also with lower life satisfaction [15], the Family Affluence Scale [18] and family structure were also included as control variables at the student level.

### Statistical method

Two-level binary logistic regression analyses were performed, presenting adjusted odds ratios (OR) with 95% confidence intervals (95% CI). Two models are presented. Model 1 includes all the student level variables, and Model 2 adds the class level variable. In addition, an empty model, containing no independent variables, was fitted in order examine the amount of variation in life satisfaction that could be attributed to the class level (i.e. between-class variation) rather than to the student level. The intra class correlation (ICC) is presented for the empty model and for Models 1 and 2. For binary outcomes this is an approximation of the amount of variation in the dependent variable that can be attributed to the higher level. In the present study, this means that the ICC indicates the proportion of unexplained variation in high life satisfaction that is accounted for by the class level.

## Results

As shown in Table 1, 84.9% of the study sample reported high life satisfaction. Further, 5.2% reported that they had been exposed to sexual jokes. The study sample was evenly distributed by gender (48.8% boys and 51.2% girls). Students in grade 5 comprised 27.0% of the sample, students in grade 7 comprised 33.3%, and students in grade 9 amounted to 39.7%. The proportion of students living with two parents was 72.4% and the mean value of the Family Affluence Scale was 9.4. The mean proportion of students in a school class who had been exposed to sexual jokes was 5.2%.

**Table 1:** Descriptive statistics and results from two-level binary logistic regressions of high life satisfaction by exposure to sexual jokes and proportion of students in class who have been exposed to sexual jokes

	Descriptives		Model 1		Model 2	
	n	%	OR	95% CI	OR	95% CI
High life satisfaction	3148	84.9				
Student level						
Exposed to sexual jokes						
No (ref.)	3519	94.8	1.00	–	1.00	
Yes	191	5.2	0.38***	0.27–0.53	0.43***	0.30–0.61
Gender						
Boy (ref.)	1810	48.8	1.00		1.00	
Girl	1900	51.2	0.52***	0.43–0.63	0.52***	0.43–0.63
Grade						
5 (ref.)	1000	27.0	1.00	–	1.00	–
7	1237	33.3	0.60***	0.45–0.80	0.62**	0.46–0.82
9	1473	39.7	0.43***	0.33–0.56	0.45***	0.34–0.60
Family structure						
Two parents (ref.)	2685	72.4	1.00	–	1.00	–
Other	1025	27.6	0.62***	0.51–0.76	0.62***	0.51–0.75
	**Mean**	**s.d**				
Family affluence	9.4	2.0	1.21***	1.15–1.26	1.20***	1.15–1.26
Class level						
% students in class exposed to sexual jokes	5.2	6.6			0.98*	0.97–0.9994
	**Empty model**					
ICC	7.3%		2.2%		1.9%	

n = 3710 distributed across 209 classes

^***^p < 0.001, **p < 0.01, *p < 0.05

The empty model indicated that 7.3% of the variation in high life satisfaction across classes could be attributed to the class level (Table 1). As demonstrated in Model 1 (Table 1), students who were exposed to sexual jokes at school had a lower likelihood of reporting high life satisfaction (OR 0.38, 95% CI 0.27–0.53, p < 0.001), whilst adjusting also for gender and grade. The ICC was 2.2%, indicating that the student level variables accounted for part of the between-class variation in high life satisfaction. Next, Model 2 (Table 1), adding the class proportion of students exposed to sexual jokes, showed a statistically significant, negative association between the proportion of students who were exposed to sexual jokes at school and high life satisfaction at the student level (OR 0.98, 95% CI 0.97–0.9994, p = 0.041), even when adjusting for the student level analogue as well as the control variables. We tested for interactions between student level and class level exposure to sexual jokes and gender and grade, respectively, but these were not statistically significant (data not presented in Table).

The class level measure of sexual jokes was unevenly distributed in that a substantial proportion of the students attended classes where no one reported to be exposed to sexual jokes. Therefore, we also constructed a trichotomous measure of sexual jokes at the class level, distinguishing classes where 0%, 1–7%, and > 7% of the students were exposed to sexual jokes (corresponding to 40.8%, 30.0%, and 29.2% of the study sample). Results from a fully adjusted two-level binary logistic regression analysis including this categorical class level measure showed a negative, statistically significant association with life satisfaction only for students attending classes with a high proportion of students who had been exposed to sexual jokes, even when controlling for exposure to sexual jokes at the student level (students in classes with 1–7% exposed to sexual jokes: OR 0.85, 95% CI 0.66–1.09, p = 0.203; students in classes with > 7% exposed to sexual jokes: OR 0.75, 95% CI 0.58–0.97, p = 0.027) (see Additional file 1: Table S1).

Finally, we also ran fully adjusted models stratified by gender and by grade (see Additional file 1: Table S2). The results showed that exposure to sexual jokes was inversely associated with life satisfaction among both boys (OR 0.35, 95% CI 0.21–0.61, p < 0.001) and girls (OR 0.50, 95% CI 0.31–0.80, p = 0.004), and among students in all grades (grade 5: OR 0.31, 95% CI 0.12–0.82, p = 0.019; grade 7: OR 0.35, 95% CI 0.19–0.64, p = 0.001; grade 9: OR 0.54, 95% CI 0.32–0.89, p = 0.016). In these stratified analyses, the association between sexual jokes at the class level and life satisfaction at the student level was in most cases negative, although the estimate was statistically significant only for grade 7 students.

## Discussion and conclusions

This study showed that students who were exposed to sexual jokes at school had a lower likelihood of reporting high life satisfaction, compared with students who were not exposed to sexual jokes. Furthermore, an inverse association was found between the class proportion of students who were exposed to sexual jokes and students’ likelihood of reporting high life satisfaction, whilst adjusting also for exposure to sexual jokes at the student level. Further analyses indicated that this association was driven by students attending classes where a relatively higher proportion of students had reported to be exposed to sexual jokes. By focusing on life satisfaction as one aspect of positive mental health [11, 12, 15–17], the findings from the present study corroborate and extend results from previous research which has demonstrated associations between psychological health problems and student level exposure to sexual harassment [1, 2, 4, 6–8], as well as with class level exposure to sexual jokes specifically [1]. To conclude, the findings point at the importance of fostering a school climate that does not tolerate sexual harassment. Such a climate may not only prevent psychological health problems, but also promote students’ positive mental health. Thus, clear rules against sexual harassment and showing disapproval when it occurs may be strategies that can assist schools to fulfil their commitment to health prevention and promotion. Lastly, while prior research has reported that school characteristics such as the proportion of students with less-educated parents, larger school and class size, and a negative school climate were associated with higher levels of sexual harassment [19], more inquiry into the school- and class-level correlates of sexual harassment is wanted. Such knowledge is highly relevant from an academic perspective, but may also support schools in their efforts to promote a favourable social climate, which can in turn strengthen positive mental health among students.

## Limitations

One limitation with the current study is that sexual jokes was measured by only one item which was dichotomised. Furthermore, the item was included in a battery of questions on bullying and it is possible that this had an impact on the students’ responses; for instance, that sexual jokes which the respondents did not define as acts of bullying were not reported. Additionally, the question offers no opportunity to determine, e.g., if the sexual jokes were made by close friends or not, by students from other classes or from the same class, and by the same gender or not. Future studies that investigate the relationship between individual and contextual expressions of sexual harassment and health outcomes should include more detailed measures of sexual harassment, preferably also using validated scales. Another important limitation is the fact that the variable measuring gender included only two categories: boys and girls. It is possible that students with gender incongruence skipped this question due to a lack of appropriate response categories. Future research should examine the links between exposure to sexual jokes and life satisfaction also within this category of students. Furthermore, the cross-sectional nature of the data should be mentioned as a limitation, since it prevented us from disentangling the temporal order of students’ exposure to sexual jokes and their reports of life satisfaction. Finally, since the study was based on data collected among students in Sweden, the generalisability of the findings may be limited. The links between individual and contextual exposure to sexual jokes and students’ positive health need to be corroborated using data collected in other societal contexts and in other age groups.

## Supplementary Information

**Additional file 1**: **Table S1**. Descriptive statistics and results from two-level binary logistic regressions of high life satisfaction by exposure to sexual jokes and proportion of students in class who have been exposed to sexual jokes (trichotomous measure). n=3,710 distributed across 209 classes. **Table S2**. Results from two-level binary logistic regressions of high life satisfaction by exposure to sexual jokes and proportion of students in the class who have been exposed to sexual jokes, stratified by gender and by grade, respectively.

## Data Availability

The Swedish HBSC data of 2017/18 can be applied for at the Public Health Agency of Sweden. Data from previous waves in Sweden and in other participating countries is available at: https://www.uib.no/en/hbscdata.

## Abbreviations

HBSC: Health Behaviour in School-aged Children
95% CI: 95% Confidence interval
ICC: Intraclass correlation
OR: Odds ratio
