# Supplementary material for: Sexual jokes at school and students’ life satisfaction: findings from the 2017/18 Swedish Health Behaviour in School-aged Children study
Source: BMC Res Notes. 2021 Jul 26;14:288. doi: 10.1186/s13104-021-05691-9 (PMC8314544; doi:10.1186/s13104-021-05691-9)
Supplement: Supplementary file 1 — Additional file 1: Table S1. Descriptive statistics and results from two-level binary logistic regressions of high life satisfaction by exposure to sexual jokes and proportion of students in class who have been exposed to sexual jokes (trichotomous measure). n=3,710 distributed across 209 classes. Table S2. Results from two-level binary logistic regressions of high life satisfaction by exposure to sexual jokes and proportion of students in the class who have been exposed to sexual jokes, stratified by gender and by grade, respectively. [file 13104_2021_5691_MOESM1_ESM.docx]

Additional file 1

Table S1. Descriptive statistics and results from two-level binary logistic regressions of high life satisfaction by exposure to sexual jokes and proportion of students in class who have been exposed to sexual jokes (trichotomous measure). n=3,710 distributed across 209 classes

|  | Model 1 | |
| --- | --- | --- |
|  | OR | 95% CI |
| *Student level* |  |  |
| Exposed to sexual jokes |  |  |
| No (ref.) | 1.00 |  |
| Yes | 0.42*** | 0.30-0.60 |
| Gender |  |  |
| Boy (ref.) | 1.00 |  |
| Girl | 0.52*** | 0.42-0.63 |
| Grade |  |  |
| 5 (ref.) | 1.00 | - |
| 7 | 0.62** | 0.46-0.82 |
| 9 | 0.46*** | 0.35-0.61 |
| Family structure |  |  |
| Two parents (ref.) | 1.00 | - |
| Other | 0.62*** | 0.51-0.76 |
| Family affluence | 1.20*** | 1.15-1.26 |
| *Class level* |  |  |
| % students in class exposed to sexual jokes |  |  |
| 0% (ref.) | 1.00 |  |
| 1-7% | 0.85 | 0.66-1.09 |
| >7% | 0.75* | 0.58-0.97 |
|  |  |  |
| ICC | 1.7% |  |

***p<0.001 ** p<0.01 *p<0.05

Table S2. Results from two-level binary logistic regressions of high life satisfaction by exposure to sexual jokes and proportion of students in the class who have been exposed to sexual jokes, stratified by gender and by grade, respectively.

|  | Boys  (n=1,810) | | Girls  (n=1,900) | | Grade 5  (n=1,000) | | Grade 7  (n=1,237) | | Grade 9  (n=1,473) | |
| --- | --- | --- | --- | --- | --- | --- | --- | --- | --- | --- |
|  | OR | 95% CI | OR | 95% CI | OR | 95% CI | OR | 95% CI | OR | 95% CI |
| *Student level* |  |  |  |  |  |  |  |  |  |  |
| Exposed to sexual jokes |  |  |  |  |  |  |  |  |  |  |
| No (ref.) | 1.00 | - | 1.00 | - | 1.00 | - | 1.00 | - | 1.00 | - |
| Yes | 0.35*** | 0.21-0.61 | 0.50** | 0.31-0.80 | 0.31* | 0.12-0.82 | 0.35** | 0.19-0.64 | 0.54* | 0.32-0.89 |
| Gender |  |  |  |  |  |  |  |  |  |  |
| Boy (ref.) |  |  |  |  | 1.00 | - | 1.00 | - | 1.00 | - |
| Girl |  |  |  |  | 0.61* | 0.39-0.95 | 0.44*** | 0.31-0.62 | 0.53*** | 0.40-0.71 |
| Grade |  |  |  |  |  |  |  |  |  |  |
| 5 (ref.) | 1.00 | - | 1.00 | - |  |  |  |  |  |  |
| 7 | 0.78 | 0.50-1.23 | 0.53** | 0.37-0.77 |  |  |  |  |  |  |
| 9 | 0.51** | 0.33-0.78 | 0.42*** | 0.30-0.59 |  |  |  |  |  |  |
| Family structure |  |  |  |  |  |  |  |  |  |  |
| Two parents (ref.) | 1.00 | - | 1.00 | - | 1.00 | - | 1.00 | - | 1.00 | - |
| Other | 0.65* | 0.47-0.91 | 0.60*** | 0.46-0.77 | 0.55** | 0.35-0.86 | 0.59** | 0.42-0.84 | 0.69* | 0.52-0.92 |
| Family affluence | 1.21*** | 1.13-1.31 | 1.20*** | 1.13-1.27 | 1.13* | 1.01-1.26 | 1.23*** | 1.13-1.33 | 1.23*** | 1.15-1.32 |
| *Class level* |  |  |  |  |  |  |  |  |  |  |
| % students in class exposed to sexual jokes | 0.98 | 0.96-1.004 | 0.99 | 0.97-1.005 | 1.00 | 0.95-1.05 | 0.98* | 0.95-0.997 | 0.99 | 0.97-1.01 |
|  |  |  |  |  |  |  |  |  |  |  |
| ICC | 2.3% |  | 1.8% |  | 4.3% |  | 0.0% |  | 2.1% |  |

***p<0.001 ** p<0.01 *p<0.05
